# Obinutuzumab Treatment in Rituximab- and Bortezomib-Resistant Phospholipase A2 Receptor Antibody-Positive Membranous Nephropathy

**DOI:** 10.7759/cureus.104285

**Published:** 2026-02-26

**Authors:** Uygar Yildirim, Sultan Ozkurt, Ahmet Ugur Yalcin

**Affiliations:** 1 Nephrology, Faculty of Medicine, Eskisehir Osmangazi University, Eskisehir, TUR

**Keywords:** bortezomib, membranous nephropathy, obinutuzumab, pla2r antibodies, rituximab resistance

## Abstract

Primary membranous nephropathy (MN) is a frequent cause of nephrotic syndrome in adults and is commonly linked to circulating autoantibodies against the M-type phospholipase A2 receptor (PLA2R). Rituximab has proven effective as a B-cell-depleting therapy in many patients; however, a significant proportion eventually develop resistance. We report the case of a 54-year-old man with PLA2R antibody-positive primary MN who did not respond to multiple immunosuppressive therapies, including cyclophosphamide, calcineurin inhibitors, repeated rituximab courses, and bortezomib. Despite ongoing nephrotic syndrome and declining renal function, treatment with obinutuzumab resulted in rapid and sustained clinical improvement. This was associated with a substantial reduction in proteinuria, normalization of serum albumin, and recovery of renal function. Our findings suggest that obinutuzumab may offer an effective therapeutic option for highly refractory PLA2R antibody-positive MN and that achieving more complete B-cell depletion could overcome resistance to conventional anti-CD20 therapy.

## Introduction

Membranous nephropathy (MN) is a primary cause of nephrotic syndrome in adults [1]. Recent advances have clarified that circulating autoantibodies against the podocyte-expressed M-type phospholipase A2 receptor (PLA2R), predominantly of the IgG4 subclass, play a key role in the pathogenesis of primary MN. Anti-PLA2R antibodies are detectable in about 70% of idiopathic MN cases and serve as important biomarkers for monitoring disease activity [2]. This recognition of MN as an immune-mediated disorder has driven a shift toward targeted therapies focusing on B-cell modulation.

The 2021 Kidney Disease: Improving Global Outcomes (KDIGO) guidelines recommend tailoring immunosuppressive therapy based on the predicted risk of disease progression. Rituximab or calcineurin inhibitors are indicated for patients at intermediate risk, while cyclophosphamide is reserved for those considered at high risk. Patients classified as low risk are usually managed with optimized supportive care [3]. Despite this structured approach, roughly one-third of patients do not achieve remission using standard regimens.

Conventional immunosuppressive therapies are often limited by treatment-related adverse effects, including infections, as well as high relapse rates. These challenges have prompted research into more selective immunomodulatory agents with improved safety profiles. Approaches that target specific components of the humoral immune response, such as B cells, plasma cells, and autoantibody production, have shown promise, particularly in cases resistant to standard therapy.

B-cell depletion is generally effective in MN, and in patients with PLA2R antibody-positive disease, reductions in antibody titers often precede improvement in proteinuria [4,5]. Nevertheless, cases refractory to rituximab, and evidence of higher remission rates with cyclophosphamide compared with rituximab, suggest that long-lived plasma cells may contribute to persistent autoantibody production in some patients [6,7]. This observation has led to consideration of plasma cell-directed therapies such as bortezomib, a proteasome inhibitor commonly used in multiple myeloma. Several case reports have described clinical and immunologic responses to bortezomib in refractory PLA2R-positive MN [8,9].

Obinutuzumab, a humanized type II anti-CD20 monoclonal antibody, has demonstrated enhanced B-cell cytotoxicity compared with rituximab in preclinical studies [10]. The NOBILITY trial confirmed that obinutuzumab induces rapid and sustained peripheral CD19+ B-cell depletion [11]. Its mechanism involves greater affinity for Fc receptors on effector cells and enhanced antibody-dependent cellular cytotoxicity, which differentiates it from type I anti-CD20 antibodies like rituximab [12]. Accumulating evidence, largely from case series, indicates that obinutuzumab can be effective in patients with MN resistant to rituximab, particularly in those with persistent PLA2R antibody positivity [13-16].

Here, we report a case of PLA2R antibody-positive MN in which rituximab-induced peripheral B-cell depletion failed to eliminate serologic activity. Bortezomib was administered based on the hypothesis that plasma cells maintained antibody production, but no response was observed. Subsequent treatment with obinutuzumab resulted in rapid clinical improvement and sustained remission.

## Case presentation

A 54-year-old male patient with preserved renal function (eGFR >90 mL/min) and no comorbidities presented in February 2017 with nephrotic syndrome and proteinuria of 25 g/day. Based on renal biopsy findings and clinical evaluation, he was diagnosed with primary MN. Complete remission was achieved with the modified Ponticelli protocol (steroids and cyclophosphamide).

In December 2017, nephrotic syndrome relapsed with proteinuria of 9 g/day, and the patient received rituximab twice (1 g on days 1 and 15), repeated six months later, resulting in complete remission. After four years, nephrotic syndrome recurred in January 2021 with proteinuria of 7.6 g/day, and complete remission was again achieved with rituximab treatment.

In November 2021, nephrotic syndrome relapsed, with proteinuria of 8 g/day. PLA2R antibody was positive (26.3 U/mL; >20 positive, <14 negative, 14-20 borderline), and the patient did not respond to subsequent rituximab repetitions or tacrolimus therapy (administered for one year with a target trough level of 6-8 ng/mL). In May 2023, the patient received cyclophosphamide (1 g/day for three doses). Corticosteroids were not administered due to bilateral femoral head avascular necrosis, and no response was observed.

The peripheral B-cell count was markedly reduced [CD19+ 4.6% (normal 6-29%)], but PLA2R antibody positivity persisted. In August 2023, based on the assumption that plasma cells might be responsible for ongoing PLA2R antibody production despite peripheral B-cell depletion, bortezomib was administered at a dose of 1.3 mg/m² (3 mg/day) on days 1, 4, 8, and 11. Pretreatment laboratory values included serum creatinine of 1.78 mg/dL, eGFR (CKD-EPI) 40.56 mL/min, serum albumin 2.49 g/dL, urine protein-to-creatinine ratio 7.8 g/day, and a PLA2R antibody level of 303.8 U/mL. Despite bortezomib therapy and maximal antiproteinuric treatment, nephrotic syndrome persisted.

Obinutuzumab treatment was initiated in September 2024 (100 mg on day 1, 900 mg on day 2, and 1 g on day 15). Pretreatment serum creatinine was 1.44 mg/dL, eGFR 52.04 mL/min, serum albumin 2.63 g/dL, and 24-hour urine protein excretion was 7232 mg/day. At the fourth month of obinutuzumab treatment (January 2025), near-complete partial remission was achieved: edema resolved, diuretic therapy was discontinued, 24-hour urine protein excretion decreased to 1566 mg/day, serum creatinine was 1.24 mg/dL, eGFR was 61.92 mL/min, and serum albumin increased to 4.1 g/dL. No adverse effects were observed during obinutuzumab treatment, and the patient tolerated the therapy well.

At one year after obinutuzumab treatment (September 2025), 24-hour urine protein excretion was 677 mg/day, serum creatinine 1.13 mg/dL, eGFR (CKD-EPI) 69.28 mL/min, and serum albumin was 4.27 g/dL, indicating near-complete remission (Table 1).

**Table 1:** Laboratory parameters before and after bortezomib and obinutuzumab therapy. PLA2R: phospholipase A2 receptor; eGFR: estimated glomerular filtration rate.

Parameter	Reference range	Before bortezomib (August 2023)	After bortezomib/before obinutuzumab (September 2024)	Fourth month of obinutuzumab (January 2025)	12th month of obinutuzumab (September 2025)
Serum creatinine (mg/dL)	0.7–1.3	1.78	1.44	1.24	1.13
eGFR (mL/min/1.73 m²)	>90	40.56	52.04	61.92	69.28
Serum albumin (g/dL)	3.5–5.0	2.49	2.63	4.10	4.27
24-Hour urine protein (mg/day)	<150	7800	7232	1566	677
PLA2R antibody (U/mL)	<14 negative	303.8	-	-	-

## Discussion

Although remission rates are generally high in MN, particularly with rituximab or corticosteroid-cyclophosphamide therapy, approximately 20-30% of patients develop refractory disease. The 2021 KDIGO guidelines do not provide a universally accepted definition for resistance, but persistent nephrotic syndrome, ongoing or worsening proteinuria, and unchanged or increasing PLA2R antibody levels after three months of appropriate therapy are suggestive of treatment resistance [3,17].

For patients failing rituximab or cyclophosphamide, enrollment in clinical trials exploring novel therapies is recommended [3]. Emerging options include next-generation anti-CD20 antibodies such as obinutuzumab and plasma cell-targeted agents like bortezomib. The rationale for these approaches is incomplete B-cell depletion or ongoing antibody production contributing to refractory disease. Clinical data on bortezomib remain limited to small case reports [8,9,18,19].

Bortezomib directly targets plasma cells, whereas rituximab depletes CD20+ B cells, sparing antibody-secreting plasma cells [20]. Prior reports have demonstrated rapid immunologic and clinical responses with bortezomib in refractory MN [8,9,18]. However, in our patient, bortezomib did not reduce proteinuria or improve renal function, representing the first reported case of non-response to this therapy in MN.

The persistence of CD19+ B cells, despite rituximab treatment, may have contributed to treatment resistance, suggesting that incomplete B-cell depletion rather than plasma cell activity could explain the lack of response. Obinutuzumab, with its deeper and more sustained B-cell depletion, may overcome this resistance, possibly by targeting B cells in lymphoid or bone marrow reservoirs [21].

Published case series support the efficacy of obinutuzumab in rituximab-refractory MN, including patients with persistent PLA2R antibody positivity [16,22-25]. In our patient, obinutuzumab alone was administered after a year without other immunosuppressive therapy, allowing a clear assessment of its effect. Limitations include the absence of serial PLA2R antibody and CD19+ B-cell monitoring; however, the observed rapid and sustained clinical remission supports its potential role in refractory MN.

## Conclusions

This case highlights obinutuzumab as a potentially effective and well-tolerated therapeutic option for patients with PLA2R antibody-positive membranous nephropathy who are refractory to multiple lines of immunosuppressive therapy, including rituximab, cyclophosphamide, calcineurin inhibitors, and bortezomib. The rapid and sustained clinical response observed in our patient suggests that the more profound and durable B-cell depletion achieved with obinutuzumab may overcome resistance mechanisms associated with conventional anti-CD20 therapies.

These findings support the hypothesis that rituximab resistance in membranous nephropathy may be related to incomplete elimination of pathogenic B-cell populations, particularly those residing in tissue or bone marrow compartments rather than solely ongoing antibody production by long-lived plasma cells. Although limited by its single-case design and the absence of serial immunologic monitoring, this report adds to the emerging evidence supporting obinutuzumab as a promising therapeutic alternative in refractory membranous nephropathy. Larger prospective studies are required to clarify its optimal use, dosing strategies, and long-term safety profile.
